# Transcatheter Patent Ductus Arteriosus Closure in Children With Different Devices and Long-Term Results

**DOI:** 10.7759/cureus.46504

**Published:** 2023-10-04

**Authors:** Kaan Yıldız, Mustafa Kir, Pinar Prencuva, Halise Z Genc, Veysel Celiktepe, Hazer E Bozyer, Yagmur D Akcura, Huseyin Bardak, Yunus S Bayam, Nurettin Unal

**Affiliations:** 1 Pediatric Cardiology, Dokuz Eylul University Faculty of Medicine, Izmir, TUR; 2 Pediatrics, Dokuz Eylul University Faculty of Medicine, Izmir, TUR

**Keywords:** child, gianturco coils, amplatzer duct occluder ii, amplatzer duct ocluder, transcatheter closure, patent ductus arteriosus

## Abstract

Introduction: With the development of transcatheter interventional techniques and the introduction of next-generation duct occluder devices, transcatheter closure has become the first treatment option for patent ductus arteriosus (PDA) in pediatric patients. In this study, we compared the effectiveness and safety of different devices for transcatheter PDA closure in pediatric patients, focusing on long-term outcomes.

Methods: A total of 235 patients aged 0-18 years who underwent transcatheter PDA closure at a tertiary care center between January 2005 and February 2020 were included. The medical records of the cases were retrospectively evaluated.

Results: The median age of the patients was 2.3 years (range: 3.5 months to 17 years), with a mean weight of 12.8 kg (range: 5.7-43.2 kg). The mean PDA diameter at its narrowest point was 2.9 mm (range: 2.2-5.1 mm). Ductal anatomy was as follows: Type A in 98 (41.7%) patients, Type E in 36 (15.6%) patients, Type C in 32 (13.5%) patients, Type F in 27 (11.4%) patients, Type D in 23 (9.7%) patients, and Type B in 19 (8.1%) patients. Arterial access was used in 138 (57.1%) patients, venous + arterial access in 58 (24.6%) patients, and venous access only in 39 (16.5%) patients. Closure was performed with Amplatzer Duct Occluder (ADO; AGA Medical Corp., Golden Valley, MN, USA) II in 151 (64.2%) cases, ADO I in 43 (18.2%) cases, and coils in 41 (17.4%) cases. The mean fluoroscopy time and mean procedural time were 10.3 ± 4.2 minutes and 41 ± 7.2 minutes, respectively. The mean radiation dose was 1364 ± 497 cGy/min. The early closure rate after the procedure was 92%, while residual shunting on the first day post-procedure was observed in 1.8% of cases, decreasing to 0.1% at the one-month follow-up. The overall procedural success rate for all cases was 96.0%. The mean follow-up duration was 9.7 years (range: 2.9-13.8 years).

Conclusion: For percutaneous PDA closure, ADO I devices are preferred for larger defects, whereas ADO II devices are prioritized for small- to medium-sized defects instead of coils.

## Introduction

The ductus arteriosus is a vascular connection present between the aorta and pulmonary artery during fetal development. After birth, it typically closes functionally within the first 12-24 hours of life. Subsequently, it closes anatomically as the ductal tissue becomes fibrous. If the ductus arteriosus fails to close after birth, it is referred to as "patent ductus arteriosus" (PDA) [1]. The incidence of isolated PDA in term infants varies within the range of 0.03-0.08%, with a higher prevalence among females compared to males [2]. PDA patients are at an increased risk of morbidity and mortality, particularly due to heart failure, infective endocarditis, and pulmonary hypertension [3]. Transcatheter PDA closure was first introduced in 1967 using the Ivadon device developed by Porstmann et al. [4].

Coils allowing closure of small ducts (<3 mm) were developed in the 1990s. The Amplatzer Duct Occluder (ADO; AGA Medical Corp., Golden Valley, MN, USA), which permits closure of larger PDAs (up to 13 mm), was first employed in 1997 and has since become a standard treatment option. Parallel to the advancement of transcatheter techniques, various devices have been developed to accommodate the closure of a wide range of ductal anatomies. For transcatheter closure of PDA, a device that is specific to the ductal anatomy and compatible with the patient's age and weight is essential [5,6].

In this article, we compared the effectiveness and safety of different devices for transcatheter PDA closure in pediatric patients over a 15-year period at a tertiary care center, focusing on long-term outcomes.

## Materials and methods

Between January 2005 and February 2020, a total of 235 patients aged 0-18 years who underwent transcatheter PDA closure at the Department of Pediatric Cardiology, Dokuz Eylül University Faculty of Medicine, were included in this study. The medical records of the cases were retrospectively reviewed. Patients with other congenital cardiac abnormalities and those with incomplete data were excluded from the study. Patients with significant additional cardiac anomalies requiring emergency surgical intervention were also excluded. Informed consent was obtained from the families of each patient. Approval for our study was received from the Ethics Committee of Dokuz Eylül University Faculty of Medicine in accordance with the principles of the Declaration of Helsinki with decision number 2021/05-18.

Indications for closure were established in patients with diagnosed PDA and continuous murmurs detected during physical examination. None of the patients had moderate to severe pulmonary hypertension. The defects were thoroughly evaluated for shape and size using transthoracic echocardiography from the parasternal short-axis and suprasternal views. Patients with irreversible pulmonary vascular disease (Eisenmenger syndrome) or those with high pulmonary/systemic pressure and resistance ratios were deemed unsuitable for closure and were not included in this study.

The procedural success, along with demographic data, clinical characteristics, angiographic parameters, and complications at the time of the procedure, were recorded for retrospective analysis. Patients who received coil closure were categorized as Group I, those who received ADO-I closure as Group II, and those who received ADO-II closure as Group III.

Devices

During the transcatheter closure procedure, three different devices were used:

Controlled-release coils (Gianturco coil) (MReye Embolization Coil; Cook İncorporated, 750 Daniels Way Bloomington, IN 47404, USA): These devices are typically used for the treatment of PDAs with a narrowest diameter of up to 3 mm. They are made of very fine fiber wires and Dacron material [7].

ADO I device (AGA Medical Corporation, Golden Valley, MN, USA): Designed with a flexible nitinol wire mesh structure, this device is attached to the ampulla region with distal retention skirts that prevent embolization. The tubular portion that sits in the aorta is 2 mm wider than the portion in the pulmonary artery. The diameter of the retention skirt is 4-6 mm larger than the main body. The device's body length ranges from 5 mm to 8 mm [8].

ADO II device (St. Jude Medical Inc., United States): Structurally similar to the ADO I model, this device consists of two equal-sized discs with a thinner waist section in the middle. The diameter of the waist section that fits into the ductus arteriosus ranges from 3 mm to 6 mm, and its length is approximately 4-6 mm. The disc diameters are 6 mm larger than the waist section. The ADO II device is developed for the closure of ductus arteriosus with a diameter smaller than 6 mm and does not contain any filling material, making it suitable for placement using smaller 4-5F catheters [9].

Procedure technique

All procedures were performed by experienced pediatric cardiologists in the field of interventional cardiology. All procedures were conducted under heavy sedation and local anesthesia. Short sheaths were placed in the right femoral artery and vein. Patients were heparinized with 50 IU/kg of heparin after sheath placement. Heparin dosage was repeated if the activated clotting time was above 200 seconds during the first hour of the procedure.

Shunt evaluation was performed by calculating the Qp/Qs ratio using blood gas measurements.

Ductal configuration was visualized using angiography with contrast injections into the descending aorta through a pigtail catheter, using the left lateral 90-degree and right oblique 40-degree angiographic positions. The ductus anatomy was assessed, and the ductal diameter was determined based on the recorded images, following the classification by Krichenko et al. [10]. The choice of device was based on the ductal anatomy, with Gianturco coils used for PDAs with a diameter of <3 mm. For PDAs with a diameter of >3.5 mm, ADO I or ADO II devices were selected based on ductal morphology.

To retrogradely cross the PDA from the descending aorta, multipurpose or right Judkins catheters were used along with a 0.038-inch guide wire or a 0.038-inch PTFE-coated hydrophilic guide wire. Coil placement procedures were performed either transarterially or transvenously in cases where coils were used. In cases where the ADO I device was employed, the guide wire was captured with a retriever catheter after crossing from the pulmonary artery to the venous side, forming an arteriovenous loop. Subsequently, the delivery system was advanced venously and passed through the PDA into the descending aorta. The retention disc of the ADO I device was initially opened in the descending aorta. The position of the device was evaluated by aortography, and then the proximal disc was opened.

In cases using the ADO II device, the guide wire was passed through the PDA, and the delivery system was positioned from the artery into the pulmonary artery. The first disc of the ADO II device was opened in the pulmonary artery, followed by the middle disc in the ductus, and the proximal disc in the descending aorta. After aortography confirmed device placement and ruled out obstruction on the aortic side, the device was released. Coil size was determined to be twice the diameter of the narrowest part of the PDA. For duct occluder devices, a device 2-3 mm larger than the narrowest PDA diameter was preferred. To verify the device position, the presence of residual shunt, and the absence of obstruction on the aortic side, an injection into the descending aorta and pressure measurement were performed 10 minutes after device deployment. In the antegrade venous approach group, obstruction in the left pulmonary artery (LPA) or descending aorta was excluded using echocardiography.

Follow-up physical examinations, ECGs, and echocardiograms were conducted 24 hours post-closure at 1, 3, 6, and 12 months and followed by annual assessments. Residual shunt, hemolysis, infective endocarditis, LPA stenosis, and descending aorta gradient were evaluated at each follow-up visit.

Statistical analysis

Statistical analyses were performed using the SPSS Statistics version 26.0 (IBM Corp. Released 2019. IBM SPSS Statistics for Windows, Version 26.0. Armonk, NY: IBM Corp) statistical package program. Descriptive statistics were presented as the number of units (n), percentage (%), median (M), minimum (min), and maximum (max) values for categorical variables and as mean ± standard deviation/median (range) for continuous variables. Data that followed a normal distribution were evaluated using the Student's t-test, while data that did not follow a normal distribution were assessed using the Mann-Whitney U test. Additionally, chi-square analysis was employed for comparing categorical data. The threshold for statistical significance was set at p<0.05.

## Results

Between January 2005 and February 2020, percutaneous PDA closure was performed on 235 patients. The median age of the patients was 2.3 years (ranging from 3.5 months to 17 years), with an average weight of 12.8 kg (ranging from 5.7 to 43.2 kg). Out of these patients, 139 (59.1%) were male, and the male-to-female ratio was 1.45. The mean PDA diameter at its narrowest point was 2.9 mm (ranging from 2.2 to 5.1 mm). There were no statistically significant differences in ductal diameters among the groups (p>0.05). The demographic data of the patients are presented in Table 1.

**Table 1:** Demographic and clinical data by patient groups ADO: Amplatzer Duct Occluder, CoA: coarctation of the aorta, LPA: left pulmonary artery, PDA: patent ductus arteriosus, M: male, F: female

Variable	Group I (coil) 41 (17.4%)	Group II (ADO I) 43 (18.2%)	Group III (ADO II) 151 (64.2%)
Age median (range) in months	11.4 (4.5-19.2)	11.8 (2.2-21.3)	11.67 (2.2-21.3)
Sex - M/F	26/15	29/14	84/67
Weight (kg) - median (range)	12.2 (6.8-28.3)	12.6 (7.2-45.7)	13.6 (3.1-9.8)
Mode of closure – n, antegrade/retrograde	0/41	43/0	28/123
Fluoroscopy time (min) - median (range)	9.2 (5-29)	8.7 (5.2-17.4)	8.6 (4.1-19.5)
Iatrogenic CoA - n (%)	0.0	1 (2.3)	2 (1.3)
Iatrogenic LPA stenosis - n (%)	0.0	1 (2.3)	1 (0.6)
PDA residual shunt - n (%)	1 (2.4)	0.0	0.0
Device embolization - n (%)	2 (4.8)	0.0	0.0
Minor complication - n (%)	3 (7.3)	2 (4.6)	1 (0.6)
Procedure success - n (%)	39 (94.2)	41 (95.3)	150 (98.7)
Follow-up (year) - median (range)	9.8 (5.3-12.7)	9.4 (4.9-13.8)	9.9 (5.4-13.6)

Ductal anatomy was as follows: Type A in 110 patients (46.8%), Type C in 32 patients (13.5%), Type E in 31 patients (13.1%), Type D in 26 patients (11%), Type F in 18 patients (7.6%), and Type B in 18 patients (7.6%) (Table 2). In terms of vascular access, 138 patients (57.1%) had arterial access, 58 (24.6%) had venous and arterial access, and 39 (16.5%) had only venous access. Among the cases, closure was achieved with ADO II in 151 patients (64.2%), ADO I in 43 patients (18.2%), and coil in 41 patients (17.4%). Statistically significant differences were not observed among groups in terms of device types used for closure according to PDA morphology (p>0.05). The mean fluoroscopy time and mean procedure time were 10.3 ± 4.2 minutes and 41 ± 7.2 minutes, respectively. The mean radiation dose was 1364 ± 497 cGy/min (Table 2).

**Table 2:** PDA morphology and measurement values according to groups PDA: patent ductus arteriosus

Variable		Group I 41 (17.4%)	Group II 43 (18.2%)	Group III 151 (64.2%)
PDA morphology - n (%)	A	25 (60.9)	20 (46,5)	65 (43.0)
	B	2 (4.8)	5 (11.6)	11 (7.2)
	C	5 (9.7)	7 (16.2)	20 (13.2)
	D	3 (7.3)	4 (9.3)	19 (12.5)
	E	4 (9.7)	3 (6.9)	24 (15.8)
	F	2 (4.8)	4 (9.3)	12 (7.9)
PDA narrowest diameter(mm) Mean ± SD		2.5 ± 0.83	3.2 ± 0.76	3.0 ± 0.95

In two cases (0.85%) where coils were used, device embolization occurred. Both embolized coils were retrieved transcatheterally, one patient underwent closure with ADO I, and the other patient required surgical intervention. In another case where ADO I was used, the device protruded into the aorta, and it was captured with a snare. Subsequently, an ADO II, which was smaller in size, was successfully placed. The early closure rate after the procedure was 92%, while on the first day post-procedure, a residual shunt was observed in 1.8% of cases, and at the first-month follow-up, it was observed in 0.1%. Mild hematomas in the femoral region developed in six patients (2.5%) during or after the procedure, all of which resolved without complications.

There were no major complications during or after the procedure. The mean follow-up duration was 9.7 years (ranging from 2.9 to 13.8 years). The procedural success rate was calculated as 94.2% in Group I, 95.3% in Group II, and 98.7% in Group III. When considering all cases, the overall procedural success rate was 96.0%. In Group II, one patient had a mild flow peak gradient of 18, mean 9 mmHg acceleration without diastolic prolongation in the descending aorta during the early period, and in Group III, two patients had mild flow acceleration without significant gradient in the descending aorta. In both groups, one patient each had mild stenosis in the LPA. In the final follow-up of all patients, there were no obstructions or significant gradients detected in the descending aorta or LPA through Doppler. In long-term follow-up, no complications such as significant residual shunt, hemolysis, or infective endocarditis were observed.

## Discussion

Since 1938, surgical interventions have been used to close PDA, and in suitable cases, the transcatheter method has been increasingly employed since 1967 [4]. Various devices are used for percutaneous PDA closure. Coils were first applied in 1992, and in Turkey, the first successful PDA closure with coils was performed by Aydoğan et al. in 1996 [7,11]. While Gianturco coils were widely preferred worldwide in the 2000s, the use of coils has decreased over time due to the introduction of new duct occluder-type devices, the use of multiple coils in large PDAs, and the risk of embolization. Therefore, there are few studies in the literature regarding the long-term outcomes of coils [12,13]. In our study, the transcatheter PDA closure procedure using coils showed a success rate of 94.2%. This result is consistent with previous reports, such as an 89% success rate in a collaborative study in Germany, a 90.5% success rate in the study by Galal et al., and a 94.6% success rate in a large series of 243 patients from our country [13-15].

When comparing the success rates of ADO I and ADO II, no statistically significant difference was observed (p>0.05). Device embolization is one of the most important complications of PDA closure. The frequency of device embolization in the literature ranges from 0% to 6%. While embolization with coils is around 4%, the risk of embolization with ADO devices is much lower, less than 1% [16-18]. In our study, device embolization occurred in two patients who had larger ductal diameters according to our measurements (3.3 mm and 3.6 mm). Both patients' devices were captured with a snare, and PDA closure with ADO I was performed in the same session. In our series, the rate of coil embolization was found to be 4.8%. Since there was no embolization in Group II and Group III, a statistically significant difference was detected between the groups (p<0.05).

The ADO device is designed in the shape of a mushroom and automatically takes its shape when deployed. It has a larger and disc-shaped structure on the aortic side. The presence of this thin disc allows the device to be anchored in the aortic ampulla. The ADO devices provide an effective and reliable method for transcatheter PDA closure. In a study involving 29 patients under the age of one, 26 of them (89.6%) had duct occlusion devices implanted. Complete closure was achieved in 73.1% immediately after the procedure and 84.6% at 24 hours. By the third month, a complete closure rate of 96.1% was achieved [19]. The ADO device has a higher success rate in achieving complete closure compared to other devices, nearly approaching 100%. The complete closure rate at six months is over 98%, and complications are quite rare [20]. In our study, the transcatheter PDA closure procedure using ADO I showed a success rate of 95.3%.

Device embolization during the release of ADO I is one of the most important complications of the procedure. Embolization typically occurs in the pulmonary artery but can also spread into the systemic circulation. In one study, device embolization occurred in three out of 209 patients. One of these cases was due to catheter manipulation after device placement, while the other two occurred spontaneously [6]. In our study, there were no cases of device embolization with ADO I. The use of ADO I in small children, especially those under 5 kg, can potentially lead to obstruction in the pulmonary artery or aorta [21]. ADO II, on the other hand, is designed to be used more safely in small children and infants. Additionally, the design of the device allows for both antegrade and retrograde placement [9,22]. In our study, ADO II was used in 151 patients (64.2%). ADO II achieved a success rate of 98.7% in the transcatheter PDA closure procedure, which was higher than other devices.

After percutaneous PDA closure, it is possible for the devices to protrude into the aorta or create stenosis in the left pulmonary artery. This condition may be associated with the retention disc of the duct occluder or the coil used being larger than the ampulla [21,23]. In our study, there was a mild flow peak gradient of 18, mean 9 mmHg in the descending aorta in one patient in Group II, two patients in Group III had mild flow acceleration without significant gradient, in one patient in Group II, two patients in Group III had mild flow acceleration without significant gradient, and there was mild stenosis in the left pulmonary artery in one patient in each group. In the final follow-up of all patients, there was no obstruction or significant gradient detected in the descending aorta or left pulmonary artery. In a study involving 62 patients with a median age of 1.2 years who underwent transcatheter PDA closure with ADO II, the residual shunt rate was 5% immediately after the procedure, 0% at the first-year follow-up, and 0% during long-term follow-up [24]. In our study, according to the echocardiographic findings on the day after the occlusion procedure, the residual shunt rate was 1.8%, which decreased to 0.1% at the first-year follow-up.

One of the primary limitations of this study arises from the lack of complete uniformity in the selection of devices, with coils up to 3 mm in diameter being utilized for ducts and ADO II being employed for PDAs of up to 6 mm in size. This has resulted in an inherent disparity among the devices used concerning ductal dimensions within the study population. Among the limitations of this study are the relatively small sample size and the retrospective data collection. Conducting more comprehensive investigations with a larger number of children and prospective data collection would provide consolidated evidence regarding the use of these devices in the pediatric population.

## Conclusions

Thanks to newly developed devices in the long-term practice of PDA closure, the use of coils has progressively decreased. For large defects, ADO I devices are preferred, whereas, for small-to-medium defects, ADO II devices have become the primary choice due to their high success rate, low-profile delivery system, and capability for rapid and secure deployment. With advancements in transcatheter interventional techniques and the introduction of next-generation ductal occluder devices, PDA closure in pediatric patients has emerged as the primary treatment option from the past to the present, depending on ductus morphology, approach route, device selection, and operator experience.
